# From oncoproteins to spike proteins: the evaluation of intramolecular stability using hydropathic force field

**DOI:** 10.1007/s10822-022-00477-y

**Published:** 2022-10-31

**Authors:** Federica Agosta, Glen E. Kellogg, Pietro Cozzini

**Affiliations:** 1 Molecular Modeling Laboratory, Food and Drug Department, University of Parma, Parco Area delle Scienze 17/A, 43124 Parma, Italy; 2 Department of Medicinal Chemistry and Institute for Structural Biology, Drug Discovery and Development, Virginia Commonwealth University, 3298-0133 Richmond, VG USA

**Keywords:** Intramolecular stability, Scoring function, LogP_o/w_, Mutations

## Abstract

Evaluation of the intramolecular stability of proteins plays a key role in the comprehension of their biological behavior and mechanism of action. Small structural alterations such as mutations induced by single nucleotide polymorphism can impact biological activity and pharmacological modulation. Covid-19 mutations, that affect viral replication and the susceptibility to antibody neutralization, and the action of antiviral drugs, are just one example. In this work, the intramolecular stability of mutated proteins, like Spike glycoprotein and its complexes with the human target, is evaluated through hydropathic intramolecular energy scoring originally conceived by Abraham and Kellogg based on the “Extension of the fragment method to calculate amino acid zwitterion and side-chain partition coefficients” by Abraham and Leo in *Proteins*: *Struct. Funct. Genet.* 1987, 2:130 − 52. HINT is proposed as a fast and reliable tool for the stability evaluation of any mutated system. This work has been written in honor of Prof. Donald J. Abraham (1936–2021).

**Supplementary Information:**

The online version contains supplementary material available at 10.1007/s10822-022-00477-y.

## Introduction

Intramolecular interactions play a central role in the polypeptide folding process leading to a protein native conformation that represents the most energetically favourable structure in a given environment [1], [2]. The analysis of the three-dimensional protein structure and the resulting protein stability can allow an understanding of its molecular function, predict a plausible mechanism of activation, and reveal new strategies for drug discovery or optimization [3], [4].

Native structure modifications can lead to conformational variations that are inherent in the protein’s mechanism of action but are also influenced by single-point alterations due to mutations. These substitutions of native amino acids with others can be characterized by different chemical and physical proprieties of the protein and alter its ligand-binding mode, function and/or overall stability [5], [6].

In order to analyse protein stability, several computational methods have been developed ranging from molecular mechanics force-field to the most recent machine-learning tools [7].However, evaluating the relative stability of proteins after mutation is experimentally difficult and can be time-consuming, especially when considering a huge number of potential mutants. The stability of mutated proteins is shown to be influenced by intramolecular interactions, the solvent-accessible surface area, and amino-acid hydrophobicity [8]. These three key aspects are taken into account within HINT.

HINT, Hydropathic INTeractions, is a program designed for quantifying hydrophobic and polar interactions between or within molecules in the biological environment [9–11]. It was motivated by the work of Abraham and Leo[12] that extended the fragment method for predictions of log P_o/w_ (partition coefficient for solute transfer in 1-octanol/water) to amino acid residues. Briefly, HINT uses these log P_o/w_ data as the basis for a forcefield describing intermolecular and intramolecular interactions.

The HINT intramolecular energy function can be rapidly calculated from the structure in terms of a score summing atom-atom interactions. In previous work, Koparde et al. [13] showed that this score can be used as a target function to guide an X-ray crystallography refinement protocol that showed significant improvement for low-resolution data (poorer than 3.0 Å), as evaluated with the global Ramachandran score and the MolProbity clashscore [14].

This work applies the intramolecular HINT force field to evaluate the stability of mutated proteins with a particular focus on the COVID-19 Spike glycoprotein whose mutations are known to modify the affinity toward the human ACE2 and/or several antibodies. The ability of the function to estimate small energy changes related to stability induced by mutations is firstly evaluated by examining a set of oncoproteins whose mutation effects on protein stability and biological behavior are widely known. In particular, the RAS-RAF-MEK-ERK signal transduction cascade is a chain of intramolecular kinase proteins that control cellular proliferation, differentiation, and apoptosis [15]. RAS proteins exist in two different conformations: a GDP-bound state (inactive) and a GTP-bound one (active)[16]. Mutations that occur in codons 12, 13, or 61 are able to alter this conformational equilibrium with a significant stabilization of the active conformation leading to a permanently activated protein and hyperproliferative disorders [17], while mutations to RAF increase its kinase activity [18]. The Intramolecular HINT scoring of this work is applied to estimate the effects of all described point mutations on conformational stability with respect to available experimental data.

## Material and Methods

### Protein Preparation

Protein structures were downloaded from the Protein Data Bank (https://www.rcsb.org/). Since not all mutated structures were available, oncoprotein and spike protein mutations were introduced into the wild-type structure using the Pymol mutagenesis module (https://pymol.org/2/). Mutated proteins were solvated in a triclinic water box in a radius of 15 Å using the TIP3P water model, neutralized and minimized in Gromacs v.2019.4 (https://www.gromacs.org/), choosing the Amber force field. Hydrogens necessary for the partition step were introduced into the topology generation.

### Protein log P Partitioning

Protein partitioning, i.e., assigning atomic hydropathic parameters to each atom, was carried out using HINT [10], [11] with the “Dictionary” option and choosing the “semi-essential” hydrogen treatment that explicitly includes polar, unsaturated, and alpha to heteroatom hydrogens. All polar and unsaturated CH_x_ hydrogens were considered as possible H-bond donors. This option treats these hydrogens as potential hydrogen bond donors without excessively diluting the hydrophobic density. Proteins were partitioned under a neutral pH solvent condition as in a normal physiological environment. The hybridized pi/lone pairs directionality vector, derived from the geometry of the atom and its attachments with tetrahedral, trigonal bipyramidal, or octahedral classes, was chosen in order to optimize the approach direction of two interaction atoms. Applying a vector focus = 1 forces a smaller and tighter cone of interaction to be scored favourably. Optimum interactions occur when explicit ion pairs and pi orbitals of polar atoms and unsaturated atoms are oriented toward suitable electron acceptor atoms, while nonpolar atoms are treated as spherical.

### Intramolecular stability scoring

The Intramolecular HINT score was calculated as a summation of hydropathic interactions between all atom pairs (∑∑b_ij_, i = 1 to N, j = i + 1 to N) – excluding those in 1–2 (bonded) and 1–3 (angle) sets – considering the solvent-accessible surface area (S), the hydrophobic atom constant (a) and the functional distance behavior for the interaction of two different atoms (R_ij_) [10]:

$$B=\sum \sum {b}_{ij}$$

$${b}_{ij}={S}_{i } {a}_{i} {S}_{j} {a}_{j} {R}_{ij}$$

Since only the heavy atoms are known exactly and hydrogen atom positions are modeled, hydrogen bonds were described as heavy atom-heavy atom distances. Above 3.65 Å the interaction was classified as Acid/Base. Positive values represent favorable binding situations such as hydrophobic-hydrophobic, hydrogen bond, acid-base, and Coulombic, while a negative value represents unfavorable interactions such as acid-acid, base-base, and hydrophobic-polar, which represents desolvation.

### Total computational cost

The minimization phase for large systems, such as the mutated RBD-ACE2 structure which consists of 12,531 protein atoms, requires 30 min of CPU time in a local HPC system, while protein partitioning and intramolecular calculation less than min of computing time.

## Results and discussion

Small protein structural variations can be related to missense mutations generated by single nucleotide polymorphisms, which is the most common human genetic alteration. These mutations, however, occur with different frequencies [19]leading to possibly altered responses to pathogens, chemical agents, or drugs, or altered structural and/or functional characteristics of encoded proteins [20]. Mutations in the Covid-19 Spike glycoprotein are often associated with higher transmissibility, high virulence, and reduced susceptibility to antibody neutralization [21]. Characterizing the stability of thus mutated proteins and their intramolecular connections are important prerequisites for quickly assessing the possible effects of such mutations on the mechanism of action or drug susceptibility.

In order to verify the sensitivity of HINT to estimate these small structural changes, we first applied it to oncoproteins, some of the best-known examples of proteins where altered structures are associated with hyperproliferative developmental disorders and cancer. RAS and RAF are intramolecular kinase proteins that control cellular proliferation, differentiation, and apoptosis [15]. RAS proteins are a small GTPase protein family [22] that exist in two different states: the GTP-bound state (active) and GDP-bound state (inactive) [16]. They possess N, K, and H isoforms; mutations at codons 12, 13, or 61 promote GTP binding and produce constitutive activation of RAS, producing uncontrolled cell proliferation [17]. RAF kinases are a family of three serine/threonine kinases proteins of which B-RAF has the highest basal kinase activity [23]. Sequence analyses of B-RAF genes have identified different mutations in the kinase domain related to this increased kinase activity[18].

All the 18 known mutations of RAS proteins were taken into account and introduced into the wildtype structure of each isoform considering both active and inactive conformations. In total, 76 mutated structures of K- and N-RAS were thus generated. Crystallographic structures of H-RAS have already revealed higher flexibility of the two adjacent regions delimiting the binding pocket known as switch I (residues 32–38) and switch II (residues 60–75) [24]. The first is a single loop while the latter consists of a loop and an α-helix. The active conformation (GTP-bound) shows two possible different orientations of these structural elements, leading to two possible states called state 1 and state 2 in a dynamic equilibrium. Since the recruitment of the effector proteins induces a shift of the conformational equilibrium toward state 2, it is described as the real active state [25]. Considering 18 possible mutations and three different conformations, a total of 57 H-RAS proteins were generated by molecular modeling in our study. Lastly, 18 different B-RAF mutations were introduced into the kinase domain to evaluate their possible effects on intramolecular stability. All mutated structures, after solvation and neutralization, were energy minimized and the Intramolecular HINT scores were calculated for each.

**Fig. 1:**
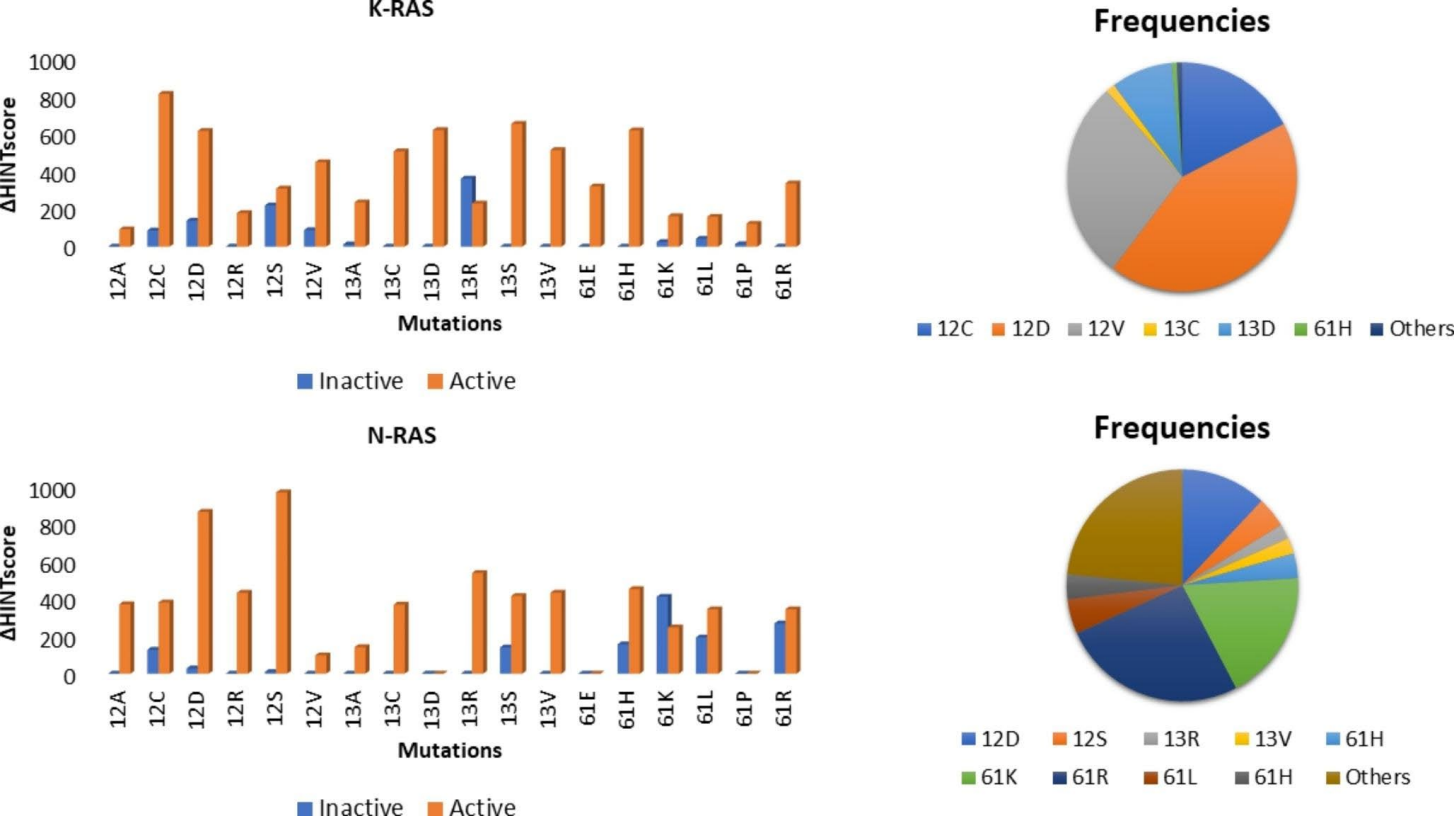
* K and N-RAS Intramolecular HINT score. K-RAS: The intramolecular stabilities of the mutated inactive (PDB ID: 5W22) and active (PDB ID: 6GOD) conformations of K-RAS were calculated. G12C, G12D, G12V, G13D, G13C, and Q61H are the most stable K-RAS closed conformations and present the highest experimental frequency. H-RAS: The intramolecular stability of the mutated inactive (PDB ID: 6WGH) and active (PDB ID: 5UHV) conformations reveal that G12D, G12S, and Q61R are the most stable and frequent N-RAS closed conformations* [16].

The mutated *inactive* K- and N-RAS conformations show minimal variations of intramolecular HINT score compared to the wildtype with a not significant ΔHINT score between mutated and wildtype form as shown in Fig. 1. However, these mutations stabilize the *active* conformations that exhibit higher intramolecular values than the wild type. So, if the intramolecular HINT score of the mutated state is higher than that of the wildtype, the active-inactive conformational equilibrium is shifted towards the *active* conformation that is *more stable* than the wildtype (Fig. 1). The stabilization of the closed conformation would thus explain the ability of high-scoring mutations to permanently activate the protein. Also of note is that the mutants with the highest experimental frequency are the most stable (Fig. 1) [16].

**Fig. 2:**
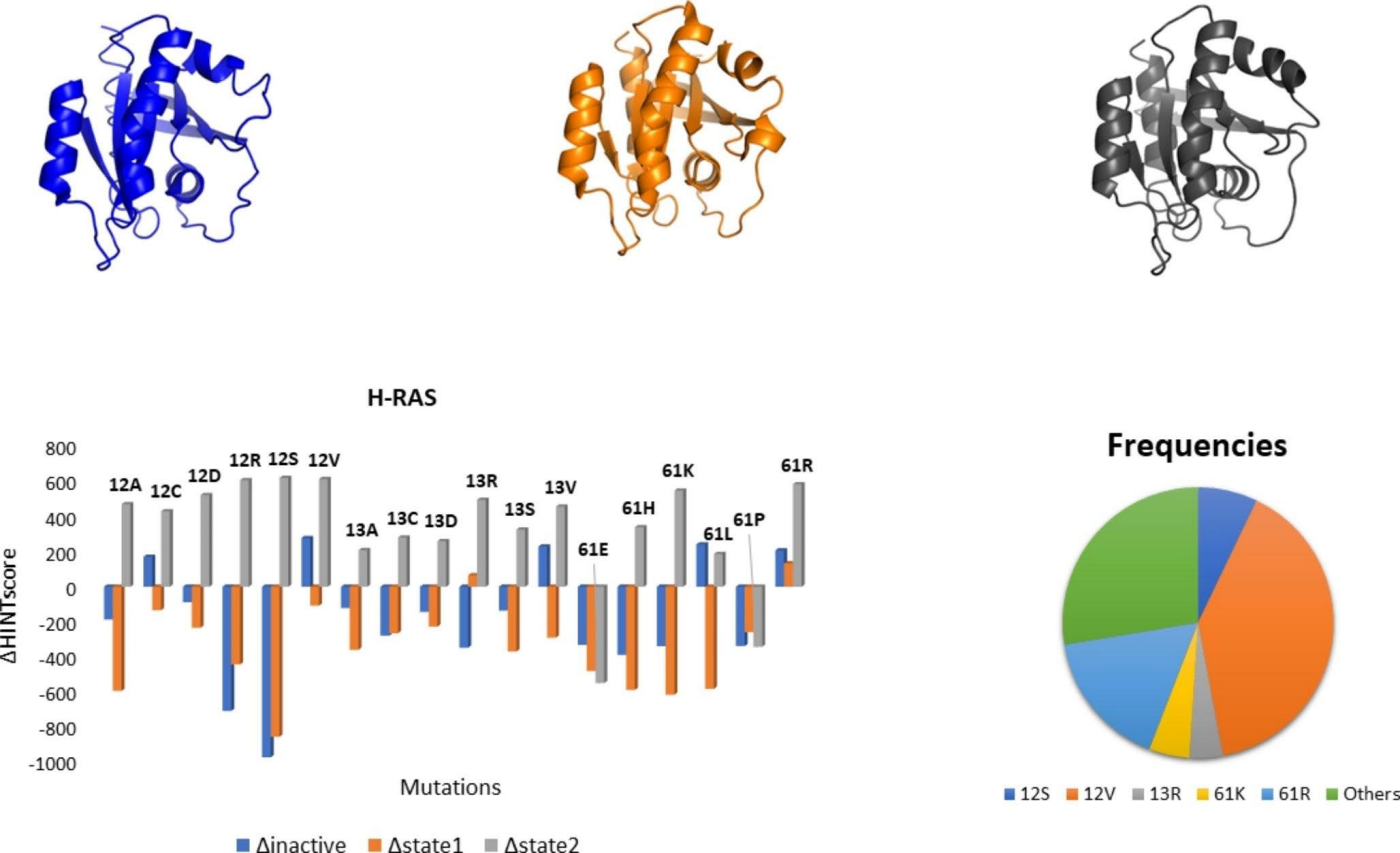
H-RAS structures and Intramolecular HINT score. Three different conformations of H-RAS were identified: an inactive GDP-bound conformation (blue) (PDB ID: 4Q21), an active state 1 conformation (orange) (PDB ID: 3RSO), and an active state 2 conformation (grey) (PDB ID: 5P21). The ΔHINT score between the mutated and the wild-type structure was calculated considering all three possible conformations. Only state 2 active conformation is stabilized by mutations

Considering the three different known H-RAS conformations, we observed that mutations destabilized the *inactive* conformations and generated a stabilization of the *active state 2* conformations. This result is in line with the explained mechanism of action of H-RAS for which only the state 2 conformation is able to recruit co-activator proteins [25]. According to our calculations, G12S, G12V, G13R, and Q61R are the most stable mutants and present the highest experimental frequency as shown in Fig. 2 [16].

Although a general stabilization of all RAS mutated active conformation is observed, different mutations produce a different effect on the intramolecular stability due to the nature of the amino-acid substitution and more complex biological mechanism that involve the residues Q61 that show a more significant conformational change when the active conformation dimerize with effector proteins [26].

**Fig. 3:**
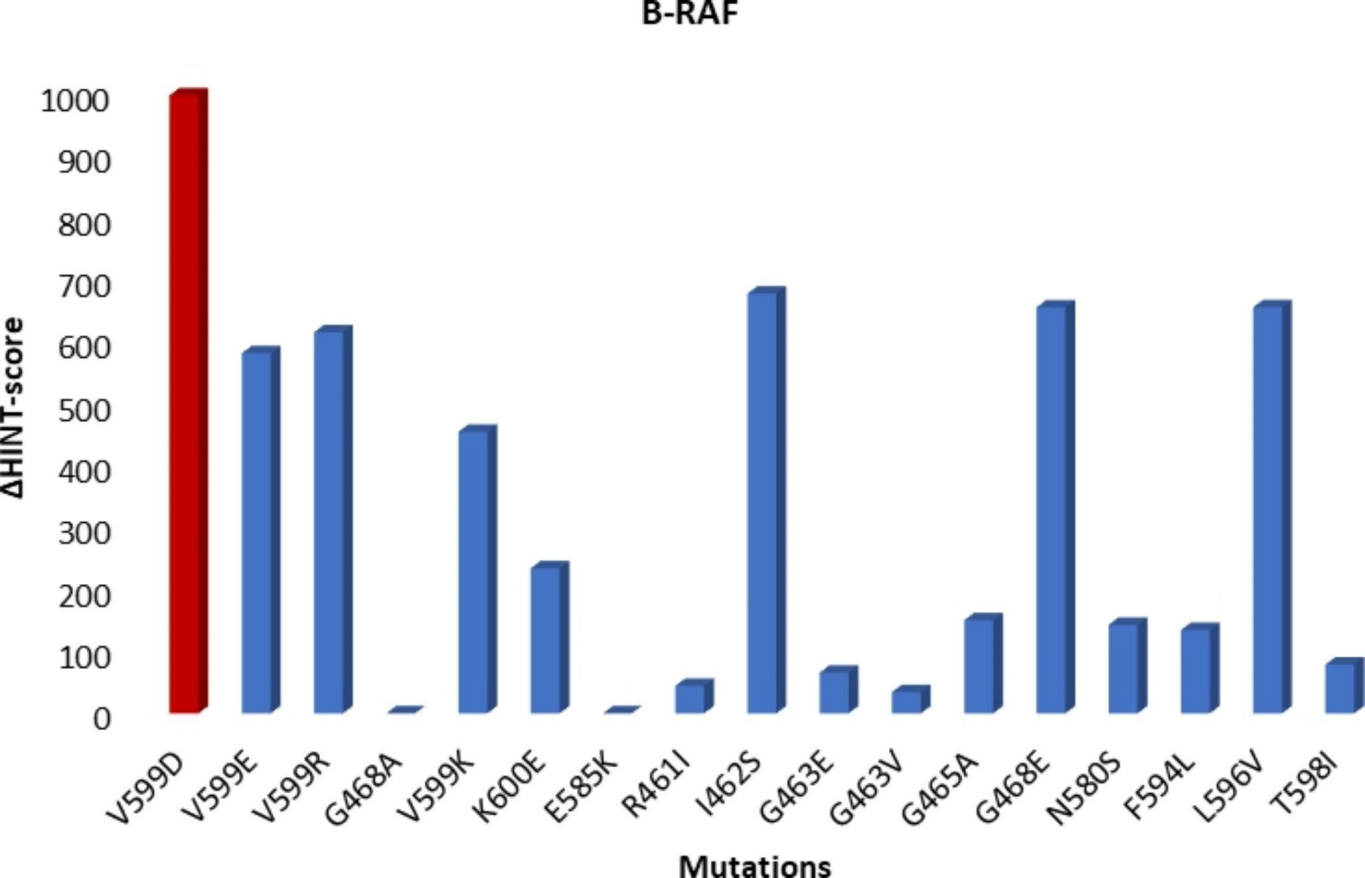
B-RAF Intramolecular HINT scores. The ΔHINT score between mutated and wildtype structure (PDB ID: 6XFP) was calculated. All mutated forms are more stable than the wild-type ones. V599D is the most stable and presents the higher experimental kinase activity

The intramolecular HINT scores reveal that all *active* mutated B-RAF proteins are as stable as, or more than, the wildtype. Wan et al. [18] describe the experimental kinase activity of B-RAS mutants. V599D is the registered mutation that most greatly increased kinase activity; our calculations also show that it shows increased protein stability as estimated by the highest intramolecular HINT score value (Fig. 3).

**Fig. 4:**
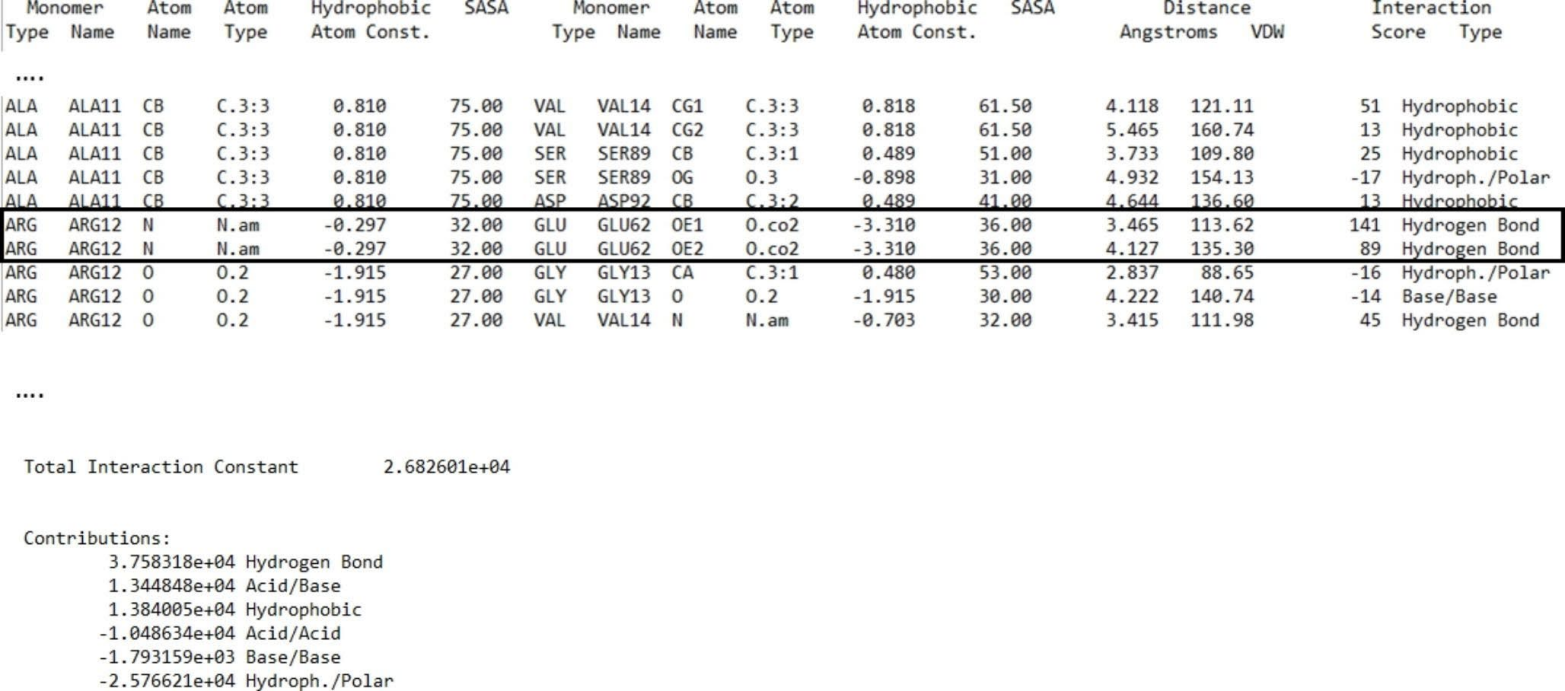
Intramolecular HINT output file. The output file shows each atom-atom interaction and the total intramolecular score with a detailed description of the single energy contribution (hydrogen bond, electrostatic and hydrophobic interactions, and negative contributions). In this capture, for example, the mutated ARG12 establishes two different hydrogen bonds with the residue GLU62.

Protein stabilization after mutation is often related to local and generally minimal structural variations that include the creation of one or more hydrogen bonds, acquisition of hydrophobic interactions, or reduction of steric bulk. The intramolecular HINT function is sensitive to each of these small changes in the protein’s energetic stability, but also records and accounts for unfavorable interactions such as changes in desolvation energy (unfavorable hydrophobic-polar score) and repulsive Coulombic interactions. In general, the intramolecular HINT function score produces results quite quickly, and these results can be correlated with experimental measurements such as demonstrated above. Also, analysis of the output on a residue pair-by-residue pair basis or even atom-by-atom basis often reveals specific information on the energetics involved in the mutation as shown in Fig. 4.

### Sars Cov2 Spike Intramolecular stability

The spike glycoprotein is a well-known and increasingly studied Covid-19 structural protein involved in virus replication due to the interaction between its receptor-binding domain and the human angiotensin-converting enzyme 2 [27]. Several mutations have been identified in different domains that can affect this interaction with the human target or lead to a reduced response to antibodies [28]. Since the spike glycoprotein is one of the main targets of vaccines, antibodies and drugs, understanding the effect of these mutations is necessary to guarantee their long-term efficacy and to monitor the clinical impact of the variant’s diffusion. Previous studies have already demonstrated that protein stability is necessary for survival and diffusion[29] by monitoring the thermodynamic effect of a single mutation on the spike/ACE2 complex stability [30]. Evaluating the effect of a single mutation could be reductive since each variant of concern is characterized by several simultaneous structural mutations. Our aim is to use the intramolecular HINT score to evaluate the stability of the known variants in the closed trimeric conformation of spike glycoprotein, the effect of mutations on the stability of the receptor-binding domain as is, and in complex with the human target.

**Fig. 5:**
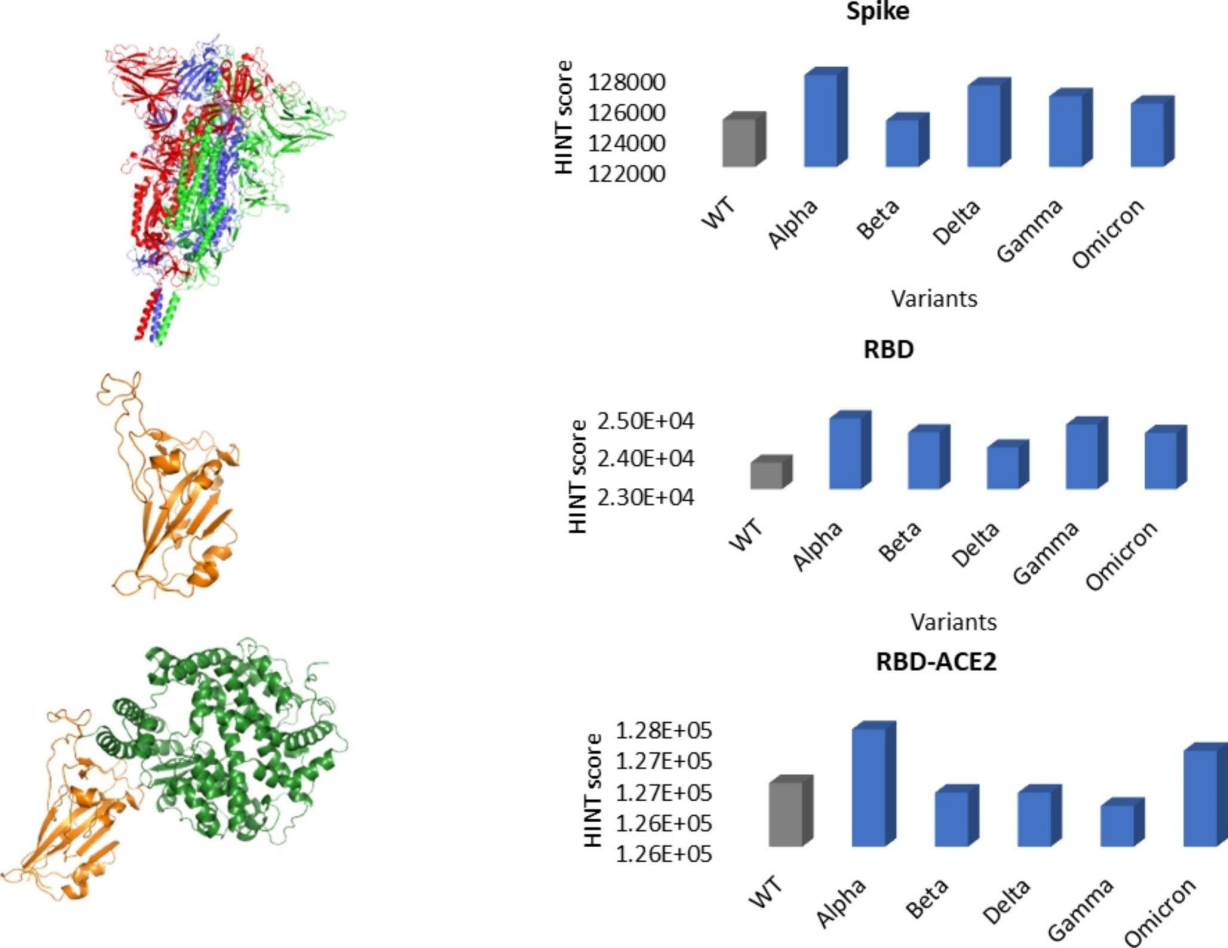
Intramolecular HINT score of Spike proteins. All mutants generated from the trimeric wildtype closed conformation (PDB ID: 6VXX) are as stable as the wild type. RBD HINT score reveals higher stability of mutants than wildtype. Alpha and Omicron variants present a higher affinity toward ACE2, and their complexes are the most stable

Alpha (B.1.1.7), Beta (B.1.351), Delta (B.1.617.2), Gamma (P.1), and Omicron (BA.1) are the principal circulating variants, defined as variants of concern by the U.S. Centers for Disease Control and Prevention (CDC) (https://www.cdc.gov/). Their structural characteristics were retrieved from GISAID [31]. Mutations were introduced in the wild-type structures and minimized. The Intramolecular HINT score evaluation shows that all mutated trimeric closed structures are at least as stable as the wildtype and all mutated receptor binding domains (RBDs) are more stable than the wildtype (Fig. 5). Alpha, Beta, and Gamma variants are characterized by one to three mutations in the receptor-binding domain with consequently minimal local variations in the structural stability. The BA.1 (Omicron) variant, despite a huge number of mutations, is stable. The Delta variant’s receptor binding domain presents a lower Intramolecular HINT score than other variants due to L452R mutation, which involves the substitution of a hydrophobic residue (leucine) with a basic and sterically bulky one (arginine) in one of the β-sheets that connect the flexible loop to the domain core.

The RBD-ACE2 complexes are all as or more stable than wildtype ones – thus underlining the variant’s higher affinity toward the human target. This effect is related to the increased local interactions generated by some substitutions like N501Y that characterized the RBD of Alpha, Beta, and Gamma variants, by acquiring a new π-π interaction. The Omicron-ACE2 complex is one of the most stable since the Omicron mutations introduce local positive charges responsible for electrostatic interaction with ACE2, which is negatively charged [32].

## Conclusion

Intramolecular HINT energy scoring allows calculating all hydropathic interactions (hydrophobic and polar) occurring within a biological macromolecule in evaluating their thermodynamic stability. It allows the evaluation of the effect of point mutations on the stability of different conformations of the same protein with a good level of agreement with the experimental data. Given its sensitivity in estimating small energy differences due to the loss or acquisition of single bonds, this function is used to evaluate the stability of the COVID_19 spike glycoprotein of the main variants of concern considering the effect of the multiple mutations occurring in the closed trimeric conformation and in their receptor-binding domain even in complex with the human ACE2.

Intramolecular HINT scoring can therefore have multiple applications and is presented as a fast, effective, and versatile tool.

## Electronic supplementary material

Below is the link to the electronic supplementary material.

Supplementary Material 1

## Data Availability

All structural data was extracted from the Protein Data Bank. All algorithms and formulas for calculations we performed and reported are presented within this manuscript and/or in reference within. Readers with questions or who wish to access HINT are encouraged to contact the program’s author, Glen E. Kellogg (gkellogg@vcu.edu).

## Abbreviations

ACE2: Angiotensin-Converting Enzyme 2
HINT: Hydropthatic Interaction
P_o/w_: Partition Coefficient between octanol and water
RAF: Rapidly Accelerated Fibrosarcoma Protein
RAS: Rat Sarcoma Virus protein
RBD: Receptor Binding Domain
SASA: Solvent Accessible Surface Area

## Declarations

### Supporting Information

The HINTscores related to all conformational protein structures evaluated in this work are provided in a .xls format: S1. RAS HINTscores (K-N and H isoforms) show the effect of all mutations on protein stability; S2. B-RAF HINTscores; S3. SPIKE proteins HINT score considering the trimeric conformation, the receptor-binding domain, and the RBD-ACE2 complexes.
